# Demystifying borderline personality disorder in primary care

**DOI:** 10.3389/fmed.2022.1024022

**Published:** 2022-11-03

**Authors:** Tina Wu, Jennifer Hu, Dimitry Davydow, Heather Huang, Margaret Spottswood, Hsiang Huang

**Affiliations:** ^1^Warren Alpert Medical School, Brown University, Providence, RI, United States; ^2^Butler Hospital, Providence, RI, United States; ^3^Duke University Hospital, Durham, NC, United States; ^4^Comprehensive Life Resources, Tacoma, WA, United States; ^5^University of Wisconsin School of Medicine and Public Health, Madison, WI, United States; ^6^Community Health Centers of Burlington, Burlington, VT, United States; ^7^Department of Psychiatry, University of Vermont College of Medicine, Burlington, VT, United States; ^8^Cambridge Health Alliance, Cambridge, MA, United States; ^9^Harvard Medical School, Boston, MA, United States

**Keywords:** borderline personality disorder, personality disorder, primary care, management of borderline personality disorder, behavioral problems

## Abstract

Borderline personality disorder (BPD) is a common mental health diagnosis observed in the primary care population and is associated with a variety of psychological and physical symptoms. BPD is a challenging disorder to recognize due to the limitations of accurate diagnosis and identification in primary care settings. It is also difficult to treat due to its complexity (e.g., interpersonal difficulties and patterns of unsafe behaviors, perceived stigma) and healthcare professionals often feel overwhelmed when treating this population. The aim of this article is to describe the impact of BPD in primary care, review current state of knowledge, and provide practical, evidence-based treatment approaches for these patients within this setting. Due to the lack of evidence-based pharmacological treatments, emphasis is placed on describing the framework for treatment, identifying psychotherapeutic opportunities, and managing responses to difficult clinical scenarios. Furthermore, we discuss BPD treatment as it relates to populations of special interest, including individuals facing societal discrimination and adolescents. Through this review, we aim to highlight gaps in current knowledge around managing BPD in primary care and provide direction for future study.

## The impact of borderline personality disorder in primary care settings: Background and epidemiology

Borderline personality disorder (BPD) is a mental health diagnosis seen in individuals who repeatedly use an array of maladaptive coping responses. This can result in unstable interpersonal relationships, mood lability, problems with impulse control, and struggles with self-image that may result in chronic feelings of emptiness and/or anger. BPD is associated with high psychiatric comorbidity, high rates of suicide, and severe functional impairment (1, 2). Up to 10% of patients with BPD die by suicide, a rate over 50 times higher than the general population (3, 4). Risk factors for suicide in BPD include comorbid depression, substance use, and posttraumatic stress disorder (PTSD) (5); inpatient psychiatric hospitalizations and lack of outpatient care (4, 6); and poor psychosocial functioning (6).

The prevalence of BPD in the general population is estimated to be between 0.5 and 2.7% (7) with a higher prevalence in specialty mental health settings (10% in outpatient psychiatry; 15–25% in inpatient) (8) and primary care (four times that of the general population and up to 19% among individuals with comorbid depression) (9, 10). In primary care, half of these patients will be undiagnosed or under-treated (9). Risk factors for BPD include a history of childhood trauma (including sexual abuse, neglect, or separation from caregivers) and family history of psychiatric disorders (11, 12). Recent family and twin studies also suggest a genetic vulnerability to BPD and evidence for a genotype-environment diathesis (13).

Individuals with BPD are more likely to have medical comorbidities such as hypertension, cardiovascular disease, and sexually transmitted diseases (14). Patients who have experienced childhood trauma from primary caregivers may be especially likely to have somatoform disorders (15). A review from 2012 found that almost 30% of those with chronic pain disorders were also diagnosed with BPD (16).

Individuals with BPD have been shown to have higher utilization of medical services, including seeing higher numbers of primary care physicians and specialists than those without BPD (17). Qualitative studies have surveyed mental health providers and emergency medicine providers regarding their attitudes to treating patients with BPD, revealing a negative personal response, greater perception of dangerousness in individuals with BPD, feelings of inadequate support or systemic resources for these individuals, and general belief that these individuals are more difficult to care for (18, 19). One small study surveying 12 primary care providers in Australia revealed that they faced similar challenges, including managing difficult behaviors and interpersonal relationships, navigating systems of support, providing accurate diagnoses, and treating medical complexities/comorbidities (20).

Both healthcare providers and patients can carry stigma around the diagnosis of BPD as individuals with BPD are frequently identified as “difficult patients” (21, 22). Strategies and support for clinicians working with patients with BPD in a variety of clinical settings have been the subject of previous articles, which we review later in this paper (23–25). Patients with BPD can be particularly difficult to work with in primary care settings, where clinicians may have limited resources, time, and experience in managing challenging or demanding behaviors. At the same time, access to mental health services can be extremely limited, especially in rural areas, which necessitates familiarity in managing BPD in the primary care setting. Given the economic and social burdens associated with BPD and the burdens of caring for such individuals by family members and clinicians, recent research has focused on early diagnosis of BPD and interventions that can be more widely disseminated (26).

Because primary care remains the entry point to treatment for many BPD patients and is the foundation of the US healthcare system, primary care providers have the opportunity to develop therapeutic, long-lasting relationships that aid in mitigating distressing and impairing symptoms. This article seeks to provide evidence-based, updated guidance for clinicians in primary care settings on the identification, engagement, and treatment of patients with BPD.

## Clinical picture

### Presentation and diagnosis

According to the Diagnostic and Statistical Manual of Mental Disorders-5 (DSM-5), individuals with BPD experience significant impairment to their self-functioning (unstable self-image or goals) and their interpersonal functioning (impaired empathy or fear of abandonment) (27). They experience negative affect (emotional lability, anxiousness, separation insecurity, or depressive symptoms); disinhibition (impulsivity or risk-taking behaviors/self-injury); and antagonism (hostility/anger). Importantly, these responses tend to be stable across time and situation and are not better explained by the individual’s developmental stage, socio-cultural environment, substance use, or other medical conditions. Individuals with BPD have also reported hallucinations (29–50%), (28, 29) delusions (20%), (28) paranoia (up to 87%), (30) and dissociative episodes (17–90%) (28, 31). Individuals who present with psychotic symptoms typically have poorer outcomes, including a two-fold increased risk for suicide attempts and higher risk for readmission to an inpatient psychiatric unit after discharge (28).

Ideally, the diagnosis of BPD should be made over time and in the absence of crises. This approach would avoid overdiagnosis and inclusion of people reverting to maladaptive coping strategies, anger, or irritability during periods of intense stress who may otherwise not meet criteria for BPD. Screening tools available to identify those with BPD include the McLean Screening Instrument for BPD (MSI-BPD) (32) and the Personality Diagnostic Questionnaire 4th edition–BPD scale (PDQ-4) (33). However, these screening tools were validated in community samples and not specifically validated for primary care settings or general medical settings. It is also important to note that in psychiatric settings, BPD is most commonly diagnosed based on recognition of a confluence of impairments/difficulties as described above rather than through screening instruments. This is an important limitation to note, as the lack of access to psychiatric specialists in primary care (either through direct consultation or collaborative care) and lack of setting-specific screening tools means that BPD remains underdiagnosed in primary care settings.

### Prognosis

While a vast majority of individuals with BPD experience improvement of symptoms, half of all patients continue to have low social and vocational functioning, potentially as a result of poor emotional regulation and histories of trauma/abandonment (34–36). The Collaborative Longitudinal Personality Disorders study followed patients for > 10 years and found that 85% of patients with BPD experienced stability for at least 12 months (37). In the McLean Study of Adult Development, which followed individuals with BPD for more than 16 years, 99% of individuals experienced stability for at least 2 years, and 78% of patients had stability for at least 8 years (38). Both studies suggested that while impulsivity improved more rapidly, emotional instability lingered (39). Additionally, completed suicide remains a concern (ranging from 8 to 10%) (36), particularly for individuals with multiple failed treatments or comorbid psychiatric disorders (e.g., depression and PTSD). Early identification and treatment are recommended to reduce patients’ suffering, improve relationships with others, develop healthy coping skills, and decrease the risk of suicide and other high-risk behaviors.

## Treatment and management

### Clinical outcomes in primary care

While clinical outcome studies for treatment of BPD in specialty programs and with psychotherapy [dialectic behavioral therapy, (40) cognitive behavioral therapy, (41) psychoanalytic therapy (41)] have been largely favorable in improving BPD symptoms, there is no significant literature focused on clinical outcomes of treating BPD in primary care. In our review of the literature, no studies were identified which examined BPD outcomes from treatment specifically implemented in primary care settings.

In the absence of outcome-based studies for treatment of BPD in primary care settings, the authors propose the following areas for consideration in providing effective care for patients with BPD in primary care. These recommendations are based on review of best practice guidelines, evidence-based psychotherapeutic principles, such as those practiced in dialectical behavioral therapy (DBT) and generalized psychiatric management (GPM), and the authors’ clinical expertise. These recommendations are meant to be applied for treatment of individuals with an established diagnosis of BPD. As mentioned earlier, the barriers to accurately recognizing BPD in primary care may limit clinician ability to adopt these therapeutic approaches for all those who may benefit.

### Team-based approach

When it becomes apparent that an individual in primary care has BPD, coordinating treatment efforts for the primary care team is crucial. For instance, having brief meetings (which can be a part of huddles, if such meeting structures are in place) to discuss management can help share data, defuse/alleviate tension among staff members, and provide ideas for a focused treatment plan. Furthermore, brief meetings of the care team can be an opportunity to prevent triangulation, a phenomenon in which treatment team members develop variable and/or conflicting attitudes about the patient, and there is no unified response to certain (often maladaptive) behaviors. Team communication should be emphasized to prevent internal team conflict and mitigate disparate or contradictory responses to the patient.

### Framework for treatment

Creating a safe environment while firmly establishing boundaries within the patient-provider relationship is critical when treating patients with BPD. However, setting boundaries in a way that simultaneously reinforces the therapeutic alliance can be challenging. We recommend establishing boundaries from the beginning, as this can help eliminate the risk of surprise and potential outrage when patients’ needs cannot be immediately met. Setting consistent expectations can also guide the clinician toward practicing equitable care. When appropriate, we recommend scheduling regular follow-up visits (e.g., monthly). This structure can help patients understand that one visit is oftentimes insufficient to share their numerous concerns and collect all pertinent information. Scheduling follow-up visits shows that patient concerns are being taken seriously and allows the conversation to be continued.

Providing psychoeducation to the patient around diagnoses and comorbidities can be helpful and allows opportunities for the patient to be actively engaged in their own care. Provider barriers to discussing the diagnosis of BPD may include fear of the patient’s response and stigma within the medical community. However, discussing the diagnosis of BPD is important because it clarifies treatment goals and acknowledges the patient’s experiences. In fact, evidence suggests that patients appreciate transparency when discussing their symptoms and the stigma they may face, emphasizing the importance of improving health literacy for this patient population (42). Furthermore, patients with BPD who later find out this diagnosis has been withheld from them often leave treatment altogether (43).

The language we use to discuss the diagnosis can present an opportunity to strengthen the therapeutic relationship with patients instead of alienating them. We recommend emphasizing that BPD reflects unhealthy or maladaptive coping strategies that have formed in response to their lived experiences rather than focusing on problems with an individual’s “personality.” Normalizing that unhealthy coping strategies are a common experience can be beneficial. However, when the predominant coping mechanisms create issues for the patient, this warrants further examination, and these mechanisms should be treated and/or addressed. This discussion can follow a tell back-collaborative inquiry approach, which emphasizes open-ended, patient-centered questions; acknowledges the complexity of medical information and provides opportunities for assessing patient understanding in a non-judgmental manner; and enhances treatment collaboration and joint decision-making/responsibility (44).

Due to the high frequency of patients presenting with both BPD and histories of trauma, adopting a trauma-informed approach can also be beneficial. When patients meet criteria for PTSD as well as BPD, it is especially important to emphasize a trauma treatment framework primarily and view BPD symptomatology as manifesting in response to significant interpersonal trauma. Principles of trauma-informed care include (1) establishing safety, (2) developing trust, and (3) respecting choice (45). The goal is to provide a safe, non-judgmental space that can be accessed in a consistent manner by the patient, who then in turn begins to trust in the constancy of support. If traumatic stress is suspected, clinical guidelines are available to help inform the treatment of PTSD in the primary care setting (46).

### Psychotherapy

Psychotherapy continues to be the mainstay of treatment for BPD, and several modalities currently exist. The most well-known of these is DBT, developed by Marsha Linehan in the late 1980s (47). DBT emphasizes problem-solving, interpersonal skills, distress tolerance, validation, mindfulness, and balancing acceptance and change (47). In its standard form, DBT consists of individual and group therapy, multiple training sessions for clinicians, and 24/7 availability of staff for providing skills coaching to patients over the phone (48). To prevent clinician burnout, a therapist consultation team is also an integral part of this model. Because these elements of DBT treatment are resource- and staff-intensive, more commonly DBT treatment is provided through engagement with an individual therapist and a DBT group only. We recommend explaining DBT as a treatment modality which helps individuals learn better strategies for managing conflict and coping with overwhelming emotions. When coupled with the normalization that all individuals can develop maladaptive coping mechanisms, this approach can promote patient engagement and reduce stigma.

Other therapy modalities that have shown potential in treating individuals with BPD include mentalization-based treatment, which focuses on understanding one’s own and others’ mental states; transference-focused psychotherapy, during which the clinician and patient explore interpersonal dynamics; and schema-focused therapy, which promotes the understanding of maladaptive patterns, including those from childhood (49–51). General psychiatric management and structured clinical management focus on providing psychoeducation and are less intensive models than DBT (50). However, they have similar outcomes related to suicidality, self-injurious behavior, and hospitalizations (52). Unlike DBT, both approaches recommend limiting contact between sessions.

### Therapeutic opportunities in primary care

Finding a therapist, specifically one trained in the specific modalities above, can be difficult. Furthermore, patients may be reluctant to engage with mental health providers. Nevertheless, certain principles from these psychotherapy modalities can be adopted by primary care staff. These include validating patients’ emotions and stressors; setting clear boundaries; and scheduling regular and time-limited appointments (53). Basic principles of mindfulness (a core component of DBT), such as observing emotions without judgment, practicing acceptance, and deep breathing, can be effective (54, 55). Some individuals have found phone apps useful in incorporating mindfulness and other elements of DBT into their lives, though research on the effectiveness of phone apps is still in its infancy (56, 57). Other individuals prefer using workbooks (The Dialectical Behavioral Therapy Skills Workbook by McKay, Wood) or websites (Now Matters Now). We also recommend learning about local or online DBT group options that patients can be referred to. Further resources are provided for patients and families at the National Alliance on Mental Illness (NAMI).

When available, patients with BPD can be referred to therapists or behavioral care managers working in an integrated model, such as Collaborative Care. The Collaborative Care model (CoCM) is an evidence-based method of treating mental health conditions within primary care, demonstrated to improve clinical outcomes (58). The CoCM team consists of a primary care physician, a behavioral health care manager, and psychiatric consultants. The intervention utilizes a registry to track and follow a population of patients, delivering measurement-based care to target specific outcomes (59). In a recent study, the telehealth Collaborative Care treatment model has shown promise for benefiting patients with BPD symptomatology in primary care (60).

### Psychopharmacology

To date, there exists little evidence for psychopharmacologic treatment of BPD and no medications have been approved for BPD by the Food and Drug Administration (FDA) (50, 61). A 2021 systematic review and meta-analysis examining pharmacological treatments for BPD showed no significant improvement in the severity of BPD symptoms from treatment with second-generation antipsychotics, anticonvulsants, or antidepressants (62). As such, medications should be used carefully with “do no harm” as a guiding principle. Benzodiazepines should generally be avoided due to disinhibition which could exacerbate impulsivity, risk of misuse and dependence, and potential lethality in overdose (63). Furthermore, comorbid PTSD would also be a relative contraindication to benzodiazepine treatment due to the lack of efficacy and incurred risks (64). While it is common practice to use psychopharmacological treatment to target symptoms (e.g., sedatives/hypnotics for sleep, alpha-antagonists for nightmares/vigilance), it is important to note that these do not treat the underlying condition, are not evidence-based treatments for BPD alone, and lead to polypharmacy. For these reasons, deprescribing, or the active discontinuation of medications through recurrent risk/benefit conversations with patients, is a useful framework to mitigate polypharmacy and reduce unnecessary prescribing (65).

**FIGURE 1:**
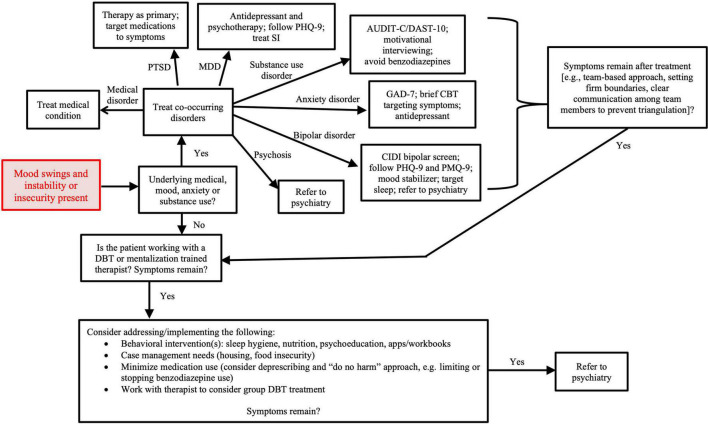
Treatment approach to mood instability in primary care. AUDIT-C, alcohol use disorder identification test-concise; CBT, cognitive behavioral therapy; CIDI, composite international diagnostic interview; DAST-10, drug abuse screening test; DBT, dialectical behavior therapy; GAD-7, generalized anxiety disorder assessment; MDD, major depressive disorder; PHQ-9, patient health questionnaire; PMQ-9, patient mania questionnaire; PTSD, post-traumatic stress disorder.

Evidence-based treatment of co-occurring disorders should be pursued. These include using a monoamine agonist (e.g., SSRI) to treat depression, anxiety, and/or PTSD symptoms. Although lamotrigine has been perceived by providers to improve affective lability, a recent study showed it was ineffective in treating individuals with BPD alone compared to placebo (66). However as discussed, it may remain an appropriate treatment if there is co-occurring bipolar disorder. Figure 1 shows the general approach for treating individuals with BPD, including identifying/treating co-occurring psychiatric disorders, providing the patient with resources, and referring to psychiatric services when appropriate.

### Managing clinician response

Oftentimes, when working with individuals with BPD, clinicians develop feelings of frustration, resentment, and hopelessness, all of which are expected, common, and valid. These may occur in response to patients who express hostility, recurrent suicidal behaviors, or other emotionally taxing interactions. Awareness of, and reflection on, these reactions to a patient and his/her behaviors are important in minimizing their interference with treatment. Grounding treatment in the knowledge that the patient struggles with maladaptive coping strategies may help clinicians stay compassionate, promote therapeutic use of boundary setting while minimizing maladaptive use of boundary setting (e.g., “punishment” for poor behaviors), and support the patient’s recovery. Processing these feelings with other peer clinicians or with a personal support network can be helpful. Table 1 provides guiding principles and examples of suggested responses to challenging behaviors that clinicians may encounter while working with patients with BPD.

**TABLE 1:** Challenging behaviors and example responses.

Problem behavior	Perpetuating response	Defusing response
Triangulation (also referred to as “splitting,” or when patients view/treat individual providers as entirely good/bad thus impacting treater relationships and potentially dividing a unified team approach)	-Taking a side -Being pulled into the enactment of the “good” and “bad” caretaker	-Take a neutral and team-based response -Educate team members and staff on a standardized and neutral approach to patient care -Establish with patient that clear communication with all treatment team members is an essential part of care and regularly coordinate treatment
Controlled substance requests, early requests, missing scripts	-Being a “helpful” and “good doctor” by granting the requests, often at the detriment of good clinical management or exacerbation of substance use disorders	-Listen and be curious -Explain clinical rationale for the prescribing/de-prescribing or not prescribing of controlled substances -Clearly describe clinic policies (including the use of controlled substance contracts) around early requests or missing scripts -Regular urine drug screens -Regular use of statewide controlled prescription awareness tools
Poor boundaries	-Ignore or accommodate the boundary violation at the expense of provider discomfort	-Firmly, yet kindly establish provider-patient boundaries
Suicidal thoughts or behaviors	-Ignore or judge the thoughts/behaviors	-Inquire about and acknowledge underlying distress -Affirm their life and your wish for them to live -Implement lethal means reduction and create a safety plan including crisis numbers/hotlines/emergency psychiatric services -Refer to mental health treatment
Non-suicidal self-injurious behaviors (NSSIB)	-Ignore the behavior -Judge or stigmatize the behavior	-Inquire about and acknowledge underlying distress -Ask about the context and purpose of the behavior (relieve or numb pain, distraction, boredom, triggers) -Discuss other strategies to release tension or cope with emotional pain (writing in journal, listening to music, holding ice, snapping hair tie against wrist) -Create a hierarchy of coping skills to keep with them
Emotionally labile outbursts, verbal abuse toward staff	-Yelling at the patient	-Gently and firmly redirect the patient -Remind them of clinic policies, treating patients and staff with respect -Inform the patient that the clinic may not be able to continue to work therapeutically with the patient if the behaviors continue
Escalating behaviors/“Upping the ante”	-Trying to take on the patient’s problems and solve them yourself	-Naming the behaviors and internal conflict to help the patient conceptualize and take responsibility for their underlying feelings
Accusing staff/providers of “not caring”	-Becoming defensive -Listing ways the patient is wrong	-Acknowledge that the patient feels uncared for and inquire what is driving that feeling -Explore the underlying wish or request that the patient has -Affirm that you care for the patient even if there is disagreement

A point worth highlighting from the table is that suicidal thoughts and behaviors are considered distinct from non-suicidal self-injurious behaviors (NSSIB), and individuals with BPD may engage in either behavior or both. The key difference between the two is that individuals who engage in NSSIB do so without intent to kill themselves but rather to relieve or distract from emotional distress. These behaviors can provoke feelings of shame and secrecy, so if not directly inquired about, patients may only discuss behaviors or thoughts directly related to suicidal intent. Despite the lack of suicidal intent, it is still important to detect and manage NSSIB as these behaviors can be harmful, dangerous, or even unintentionally lethal (67). We suggest directly asking individuals if they are engaging in behaviors to hurt themselves (cutting, hitting, burning, scratching, etc.) in response to negative emotions. In population samples (not just individuals with BPD), prevalence rates of NSSIB are highest in adolescents (7.5–46.5%) and university students (4–23%), with onset most often occurring in younger adolescence (68). Therefore, our primary colleagues, as the first point of medical contact for most individuals, are uniquely positioned to provide earlier recognition and intervention for these behaviors.

## Bias, stigma, and special populations

### Experiences of bias by patients and caregivers

As mentioned earlier, there is deep-rooted stigma around the diagnosis of BPD within healthcare settings. Acknowledging this stigma is important as it can significantly impact clinician-patient relationships and subsequent treatment. Studies suggest that patients with a diagnosis of BPD frequently feel their complaints are not taken as seriously and that they are more often negatively treated compared to individuals without this diagnosis (69). For clinicians, there can be a tendency to attribute high-risk behaviors (e.g., self-harm) to a patient’s desire for attention instead of a belief that these behaviors are a reflection of mental illness (69). This pattern of thinking can give rise to further stigmatization by conceptualizing patients as manipulative and “in control” of their behaviors, leading to reduced empathy and avoidance in treating these individuals (69). Both patients and clinicians experience pervasive feelings of powerlessness and have low expectations for recovery (in part due to a lack of adequate resources), contributing to a self-fulfilling prophecy (69).

Due to high rates of stigmatization of BPD in clinical and broader societal settings, individuals with this diagnosis commonly self-stigmatize, developing low self-esteem and feelings of helplessness (70). Patients describe being referred to as liars and manipulators and not feeling as “human” as others (71). When asked directly, individuals with BPD identify connections with others, a focus on their strengths, and the adoption of a holistic view of patients’ lives (“seeing someone as human”) as helpful (71).

Caregivers of patients with BPD have similar experiences when interacting with the healthcare system, including not having concerns about these patients taken seriously, frustration in not being able to access resources, and encountering mental health clinicians with poor health literacy/understanding of BPD (72). Caregivers have endorsed impaired well-being, interpersonal difficulties, anxiety/depression, and secondary trauma symptoms (e.g., after witnessing self-injurious behaviors) and have reported higher rates of grief and burden compared to caregivers for individuals with other serious mental illnesses (26, 73). Qualitative studies suggest caregivers often hope to be more involved in partnering with clinicians (72).

### Populations experiencing societal discrimination

A discussion of bias would be remiss without further exploration of minority groups and whether certain populations are under-or over-diagnosed. Recent studies suggest sexual minority individuals were more likely to be diagnosed with BPD compared to heterosexual individuals even after controlling for presenting symptoms, though the reason(s) for this bias remain unclear (74). In discussing the diagnosis of BPD in sexual minority populations, Rodriguez-Seijas et al. stress the effects of marginalization and discrimination and challenge our conceptualization of BPD and other personality disorders in this broader context (e.g., interpersonal difficulties better explained due to differences in culture), (74) a perspective that could likely be applied to other minority groups. Women are also more likely to be diagnosed with BPD despite recent data suggesting similar prevalence in women and men (75), which is speculated to be a result of differences in expression/recognition of BPD, gender biases when diagnosing, and sampling bias (76). Furthermore, men receive less lifetime psychotherapy and pharmacotherapy, despite similar duration of treatment (77). This may speak to a difficulty in recruiting men for BPD research samples which results in under-study, under-recognition, and under-treatment for men in particular.

Unfortunately, the prevalence, risk factors, and management of BPD in low-income, under-resourced, and ethnic/racial minority populations are under-studied. In one urban primary care study of predominantly Hispanic individuals, those who screened positive for BPD reported a high percentage of interpersonal trauma (83%), and a large majority (91%) also met criteria for a comorbid psychiatric condition (2). In another study of individuals with BPD with risk factors for poor psychosocial outcomes and suicidality over time, racial minority populations (primarily Black in this sample) were significantly associated with lower socioeconomic functioning and increased suicide risk (78). The study found that discrimination with regards to educational and employment opportunities potentially mediated this suicide vulnerability. Evidence suggests that Black adults may have different experiences of BPD (e.g., higher rates of emotional dysregulation and fewer suicidal behaviors) compared to White adults, raising concerns as to whether certain racial/ethnic minority populations are under-diagnosed and thus under-treated (79, 80).

As with most research undertakings, efforts should be made to recruit more racial/ethnic and/or sexual minority patients in studies regarding BPD. Understanding BPD in the context of minority stress [especially given high rates of comorbidity with trauma disorders (81)] and cultural differences remains an understudied area and would likely deepen our conceptual understanding of personality disorders.

### Adolescents

Controversy has existed in diagnosing BPD prior to adulthood. Opponents argue that the diagnosis should not be given when unique developmental changes and fluid personality traits influence presentation before adulthood (82, 83). However, proponents posit that temperament studies have shown personality traits tend to remain relatively stable from childhood to adulthood and therefore appropriate diagnosis can be made and lead to earlier initiation of treatment (83). In general, the current literature supports that BPD is a reliable and valid diagnosis in adolescents (84, 85). From the available epidemiological data, BPD is present in around 3% of the general adolescent population, though this is not consistent across different samples (86). Similarly to adults, evidence-based treatment centers around supportive psychotherapy (86) and manualized treatments including DBT (87), mentalization-based therapy (88), and cognitive analytic therapy (89). Pharmacological interventions, particularly the use of benzodiazepines, are not recommended for treatment of BPD alone (86). Some have advocated for “clinical staging” to identify at-risk youth and the subsequent use of appropriate interventions (e.g., psychoeducation and supportive counseling for mild/non-specific symptoms versus case management and time-limited psychotherapy after formal diagnosis of BPD) (90).

## Future directions

Over the past few decades, BPD prevalence, diagnosis, and management in primary care settings has been written about and discussed with great interest (9, 53, 91, 92). Despite this interest, there exists a real dearth in observational or interventional research studying treatment outcomes of BPD patients in primary care settings. Part of the challenge in pursuing this research is due to difficulty identifying these patients in primary care settings. Screening tools, such as the MSI-BPD and PDQ-4, have not been specifically validated in primary care or general medical settings, and primary care colleagues are unlikely to be familiar with or comfortable using these tools to aid in BPD diagnosis. In psychiatric settings, these tools have been shown to be effective at not only screening in BPD patients but also differentiating it from bipolar disorder, a commonly confused diagnosis (93). Thus, research opportunities exist in validating similar screening tools in primary care and identifying appropriate populations or triggers for screening.

With the rise in telehealth care during COVID-19, attention has been increasingly directed toward its potential benefits for treating individuals with BPD. Several studies have been published over the last year examining telehealth delivery of services to these patients in various settings, including outpatient psychotherapy (94, 95), partial hospitalization programs (96), and correctional settings (97). Telehealth has the potential to increase access to mental health treatment in primary care settings through models such as collaborative care and integrated care. We believe telehealth can provide more effective utilization of mental health care partners and care managers in primary care when managing patients with BPD and highlight this as an important area for outcomes research.

Relatedly, there has been an explosion in smartphone apps marketed toward mental health. A 2020 systematic review and meta-analysis included review of 10 smartphone apps targeting BPD symptoms. The systematic review described mixed effects of the intervention outcomes, and meta-analysis on seven randomized controlled trials ultimately revealed no significant difference in BPD-related symptoms with or without smartphone app use (98). The authors also found that most app studies included in their review did not report on serious adverse events over the course of participation. Unfortunately, there are currently no apps with a strong evidence base that we can recommend for improving BPD symptoms. Although apps may be useful in tracking moods/behaviors over time and introducing/encouraging the use of coping skills, one wonders whether these apps can provide enough of the interpersonal qualities that other interventions (e.g., psychotherapy) offer. Additionally, recent studies have suggested that while app installation rates may be high, the majority of patients do not continue using apps for long periods of time, (99) calling into question whether there can be sustained improvements. These areas remain worthy of future study and development.

Other novel areas of study are being considered and will hopefully allow us to better conceptualize BPD and understand why certain treatments may work better for certain individuals (100). A neuroscience approach to studying BPD can offer additional understanding of BPD pathophysiology over traditional psychological or behavioral approaches, which may lead to further targets for treatment (101). For example, a 2019 systematic review and meta-analysis revealed overall cortisol level differences in individuals with BPD, suggesting a disruption to the hypothalamic-pituitary-adrenal axis in BPD pathophysiology (102). However a balance should be struck between funding basic science research and clinical implementation. Beyond focusing treatment on just the individual, engaging close relationships (e.g., family, significant others) has been shown to effectively reduce BPD symptoms and emotional dysregulation (103). This family and systems-oriented treatment approach could be uniquely capitalized upon within primary care, as multiple members of a family/social network may already be engaged in the same clinic.

Lastly, concerns have been raised that funding for BPD is significantly less than for other mental health disorders. Between 1990 and 2014, the total National Institutes of Health funding for BPD was 55 million dollars, a number drastically less than the 622 million dollars spent researching bipolar disorder (104). The reason for this disparity is multifactorial and includes lack of examination/emphasis on the economic costs of BPD on society, (105) inadequate training and education for psychiatric clinicians, (106) stigma and decreased willingness to engage/study BPD, (106) and decreased advocacy (105, 107). This may be another broadly systemic reflection of bias against BPD, and we would recommend increasing both funding and psychoeducation.

## Conclusion

Patients with BPD frequently present to primary care and are often under-diagnosed and/or under-treated. The medical and psychiatric treatment of these individuals can be challenging as BPD symptoms contribute to high-risk behaviors, high psychiatric comorbidity, and impairments in interpersonal functioning. Additionally, patients are not always willing to engage in treatment and when they are, resources for treating BPD within both primary care and mental health clinics are often limited (e.g., availability of consistent psychotherapy). Training provided for using/interpreting screening tools and understanding clinical presentation could increase appropriate recognition of BPD. However, there is currently insufficient evidence supporting general screening for BPD in primary care settings, and more research is needed to validate and understand the appropriate use of these screening tools. While there are sparse clinical outcomes data to inform best treatment of BPD in primary care settings, we recommend several guiding principles to improve primary care management of patients with BPD: validate distress, maintain clear boundaries, communicate regularly with all members of the patient’s treatment team, schedule time-limited but regular appointments, and incorporate psychotherapeutic elements into the patient’s care. Psychotherapy, specifically DBT, is the mainstay of treatment and there are no FDA approved medications for the treatment of BPD alone. When faced with emotionally difficult clinical situations arising from the care of individuals with BPD, it is important for primary care clinicians to identify their own peer and personal support networks. Additional study is warranted to examine the treatment experiences and outcomes in adolescents and understudied populations (e.g., low socioeconomic, ethnic/racial minority, and sexual/gender minority populations). Future directions for study include observational/interventional outcome studies for treating BPD in primary care, integration of telehealth, validation of evidence-based apps, understanding mechanisms of change/improvement, and targeting novel areas of treatment.

## Author contributions

All authors have participated in the imagining, drafting, and revising of this project. All authors have read and approved the final submitted version of this manuscript.
